# Prognostic characteristics of a six-gene signature based on ssGSEA in sarcoma

**DOI:** 10.18632/aging.205443

**Published:** 2024-01-17

**Authors:** Jun Liu, Jianjun Lu, Gefei Wang, Liming Gu, Wenli Li

**Affiliations:** 1 Department of Clinical Laboratory, Dongguan Hospital of Guangzhou University of Chinese Medicine, Dongguan 523005, China; 2 Guangdong Provincial Key Laboratory of Infectious Diseases and Molecular Immunopathology, Shantou University Medical College, Shantou 515000, China; 3 Department of Quality Control and Evaluation, First Affiliated Hospital of Sun Yat-sen University, Guangzhou 510080, China

**Keywords:** sarcoma, overall survival, ssGSEA, MYC targets, gene signature

## Abstract

Background: Sarcoma is a rare malignant tumor originating of the interstitial or connective tissue with a poor prognosis. Next-generation sequencing technology offers new opportunities for accurate diagnosis and treatment of sarcomas. There is an urgent need for new gene signature to predict prognosis and evaluate treatment outcomes.

Methods: We used transcriptome data from the Cancer Genome Atlas (TCGA) database and single sample gene set enrichment analysis (ssGSEA) to explore the cancer hallmarks most associated with prognosis in sarcoma patients. Then, weighted gene coexpression network analysis, univariate COX regression analysis and random forest algorithm were used to construct prognostic gene characteristics. Finally, the prognostic value of gene markers was validated in the TCGA and Integrated Gene Expression (GEO) (GSE17118) datasets, respectively.

Results: MYC targets V1 and V2 are the main cancer hallmarks affecting the overall survival (OS) of sarcoma patients. A six-gene signature including VEGFA, HMGB3, FASN, RCC1, NETO2 and BIRC5 were constructed. Kaplan-Meier analysis suggested that higher risk scores based on the six-gene signature associated with poorer OS (*P* < 0.001). The receiver Operating characteristic curve showed that the risk score based on the six-gene signature was a good predictor of sarcoma, with an area under the curve (AUC) greater than 0.73. In addition, the prognostic value of the six-gene signature was validated in GSE17118 with an AUC greater than 0.72.

Conclusion: This six-gene signature is an independent prognostic factor in patients with sarcoma and is expected to be a potential therapeutic target for sarcoma.

## INTRODUCTION

Sarcomas are a type of malignant tumors originating of mesenchymal or connective tissue that occur mainly in the trunk, extremities and retroperitoneum. Sarcomas have more than 100 different histological subtypes, accounting for about 1% of all adult malignancies [1]. The overall prognosis of sarcoma is poor [2]. Factors affecting the prognosis of sarcoma patients include tumor location, size, grade, stage, metastasis, and response to chemotherapy. Among them, the grade of the primary tumor has a significant impact on the prognosis [3]. In terms of tumor site, the prognosis of axial osteosarcoma is poor compared to that of limb sarcoma. In terms of tumor grade, high-grade sarcomas are more likely to spread to other parts of the body than low-grade sarcomas, resulting in a worse prognosis. With the development of genomics, the molecular typing of sarcoma has become an important prognostic factor. Next-generation sequencing technology can detect genetic alterations in sarcomas, providing new opportunities for accurate diagnosis and treatment of sarcomas [4, 5].

As a transcription factor, the proto-oncogene MYC is considered to be the main driver of various malignant tumors. In previous studies, the MYC oncogene was found to be overexpressed in a variety of cancers and associated with poor outcomes. A number of cancers exhibit enhanced invasiveness caused by the c-MYC oncogene, and it plays an essential role in cancer progression [6, 7]. It is worth mentioning that the oncoprotein-regulated transcriptional mechanism of the MYC family may promote carcinogenesis independently of changes in the expression of its target genes [8]. Therefore, down-regulating the transcription of the oncogene c-MYC may be a feasible cancer treatment strategy [9]. Targeting the function of the MYC oncoprotein is expected to realize new and effective anticancer therapies that can be applied to a variety of cancers. However, the uncharacteristic structure of MYC protein has severely hindered the development of MYC-targeted therapy [10]. The development of c-MYC inhibitors has always been an elusive goal in the field of cancer therapy [11].

In this study, using the Cancer Genome Atlas (TCGA) database, we examined the correlation between cancer hallmarks and sarcoma patient prognosis. Using important prognostic genes associated with key cancer hallmarks, we established a prognostic gene signature. TCGA and Gene Expression Omnibus (GEO) training and validation sets were then used to validate the prognostic value of the gene signature. Moreover, the correlations between risk scores and tumor immune microenvironment, including immune cell infiltration profiles, immune function, and immune score, were investigated. Finally, a nomogram for clinical practice was built. To conclude, this study comprehensively analyzes the prognostic values of a new gene signature. As well as being used as a prognostic biomarker, this gene signature might also serve as an immunotherapy target for sarcomas.

## MATERIALS AND METHODS

### Preparation and analysis of data sets

Datasets for training and validation were obtained from the TCGA and GEO databases, respectively. There were 256 sarcoma patients in the training dataset, whose mRNA expression profiles were obtained from the TCGA database (http://cancergenome.nih.gov/). There were 60 sarcoma patients included in the validation dataset (GSE17118) containing sarcoma mRNA expression profiles and clinical information downloaded from the GEO database (http://www.ncbi.nlm.nih.gov/geo/). In addition, we converted TCGA FPKM data to TPM format for comparison between platforms. Data from the microarrays downloaded from GEO were normalized using the R package “limma” [12]. Subsequently, chip probes were mapped to gene symbols using the R package GEOquery. To obtain gene expression profiles, we removed probes that map to multiple genes and took the median of multiple probes that map to a single gene. In light of the fact that both of the above databases are public, the local ethics committee does not need to approve this study.

### The selection of candidate genes and the establishment of a gene signature

On the basis of transcriptome profiling data and hallmark gene sets from the Molecular Signature Database (MSigDB), the single-sample gene set enrichment analysis (ssGSEA) algorithm was used to quantify the performance of cancer hallmarks by using R package “gsva” in the training set [13, 14]. The principle of ssGSEA is to sort the gene expression data of each sample in order of expression from highest to lowest, and then calculate the cumulative distribution function (CDF) of the genes in each gene set in the sorted gene list. Next, the CDF of each gene set is compared to a reference distribution to obtain a standardized enrichment score (NES). NES reflects the relative enrichment degree of gene set in the sample, the larger the gene set is, the more enriched it is, and the smaller the gene set is, the sparser it is. The advantage of ssGSEA is that it can be used to assess the degree of enrichment of gene sets in different samples, as well as the degree of enrichment of gene sets under different biological conditions. Therefore, ssGSEA has been widely used in bioinformatics [15, 16]. Various cancer hallmarks in sarcomas were assessed by using the R package “survival” to conduct a univariate Cox proportional hazards regression analysis. Scale-free co-expression networks were constructed using the R package “wgcna”. Gene ssGSEA modules associated with MYC targets V1 and V2 were identified using transcriptome profiling data and scores [17]. In MYC targets V1 and V2, gene significance was utilized as a means of quantifying the correlation between individual genes and ssGSEA scores. A module member is a correlation between a gene’s expression profile and its module-characterized gene. A GS marker threshold of 0.0001 and a Cox regression *p*-value of 0.01 were used to identify 147 prognostic genes most associated with MYC targets V1 and V2 [18]. Using random forest, we ranked genes based on their importance and selected the top ten most influential genes. From multiple combinations of ten genes, a gene signature with a small number of genes and a significant *p*-value was chosen.

### Survival analysis based on risk scores

ssGSEA scores were normalized using the Z-score method [19]. Survival analysis was carried out using Kaplan-Meier method. Each parameter’s importance to overall survival was assessed with a Cox proportional hazards regression model. Patients with sarcoma were categorized into high- and low-risk groups based on their median risk score. Following that, the prognosis of the two groups was compared in the training and validation sets. ROC curves and C-indices were then used to assess the risk model’s accuracy. Additionally, a two-way survival analysis combining risk scores and cancer hallmarks Z-scores was conducted to determine the impact of the two scores on the prognosis of SARC patients. Low-risk patients with low MYC targets V1/V2 Z-scores were categorized as low-risk, while high-risk patients with high MYC targets V1/V2 Z-scores were classified as high-risk. Other patients were defined as the other group. Survival differences between groups were calculated by Kaplan-Meier curves. Furthermore, the risk model’s ability to predict survival in various subgroups in relation to age and gender was examined.

### Therapeutic drug prediction based on risk scores

By referring to previous literature, this study identified drugs with different therapeutic predictions between high-risk and low-risk groups. Specifically, the study grouped patients by analyzing their risk scores and then predicted which drugs would be most effective for a particular group of patients. This approach could help doctors better choose treatment options, improve treatment effectiveness and reduce unwanted drug side effects [20].

### Analysis of six hub genes for drug susceptibility

To explore potential targeted drugs available for the six hub genes, drug sensitivity analyses were conducted. Potential targeted drugs for these six hub genes were explored referring to the previous literature [21]. A number of R packages were used for data processing and visualization, including “impute”, “limma”, “ggplot2”, and “ggpubr”.

### Analysis of GO and KEGG enrichment

We used *p* < 0.05, |log2FC| >1 as a threshold to identify genes that are associated with high risk and low risk. These differentially expressed genes were then analyzed using GO and KEGG enrichment analysis.

### Establishment and evaluation of the nomogram in patients with sarcoma

Nomograms are effective methods for predicting cancer prognoses in clinical practice [22]. An overall survival probability for sarcoma patients was assessed with a nomogram based on a six-gene signature. Then nomogram survival predictive power was assessed using ROC curves and C-index analysis. To verify the accuracy of the nomogram, calibration curves were used to compare predicted probabilities to actual probabilities.

### Risk score and tumor immune microenvironment (TIME) correlation analysis

Considering TIME’s importance for tumor cell growth, we correlated the risk score with TIME. Firstly, the correlation between risk score and immune cell infiltration status was examined [23]. There are a number of immune cells that are involved in the analysis, including aDC, B cells, cytotoxic cells, DC, eosinophils, iDC, mast cells, NK cells, pDC, T cells, Tcm, Tgd, and Th2 cells. Also explored was the relationship between the risk score and immune function. In addition, immune scores for sarcomas were calculated using the ESTIMATE algorithm [24].

### Statisticians and bioinformaticians

Based on MSigDB hallmark gene sets, GSEA analysis was conducted to examine MYC targets V1 and V2 in high- and low-risk score groups [25]. The data analysis and graphing were carried out using IBM SPSS Statistics 23 (IBM Corp., Armonk, NY, USA) and the R programming language (version 3.5.2, http://www.r-project.org). The ssGSEA scores were normalized using Z-scores. The Kaplan-Meier method was used to draw survival curves, and the log-rank test was used to assess differences between groups. Using Cox proportional hazards regression models, the importance of each parameter was assessed. Risk scores were compared using Wilcox test.

### Availability of data and materials

The datasets analyzed in the current study are available in the TCGA repository (http://cancergenome.nih.gov/) and the GEO (https://www.ncbi.nlm.nih.gov/geo/).

## RESULTS

### Diagram of the research design

**Figure 1:**
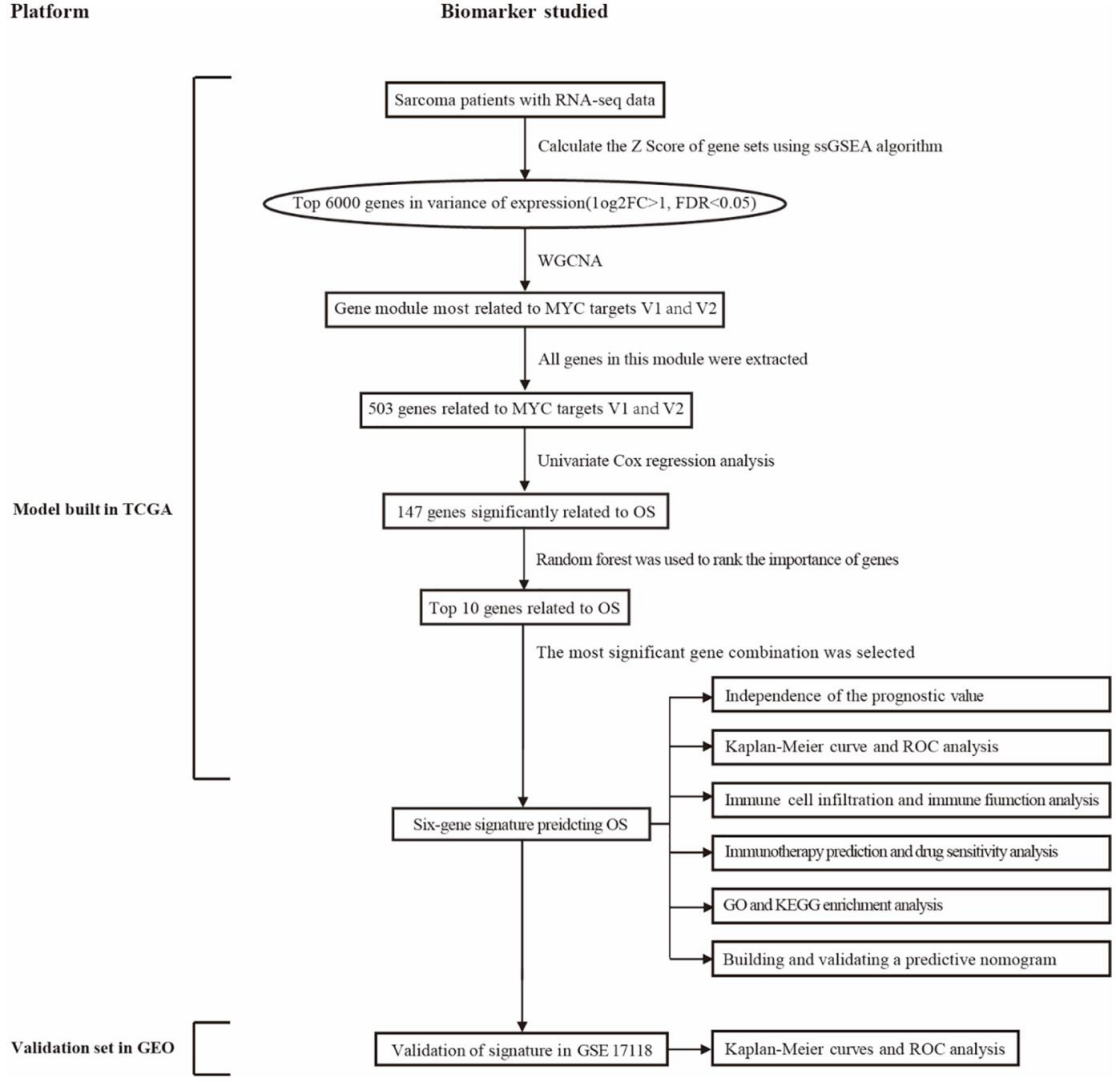
**An overview of this study’s flowchart.** Abbreviations: WGCNA: weighted gene co-expression network analysis; ssGSEA: single sample gene set enrichment analysis; GO: gene ontology; KEGG: Kyoto encyclopedia of genes and genomes; OS: overall survival; ROC: receiver operating characteristic.

Figure 1 illustrates the entire study workflow. Detailed procedures for constructing survival prediction models for sarcoma patients are provided below. As a first step, cancer hallmarks were identified as major risk factors for survival in patients with sarcoma, including MYC targets V1 and V2. Then ssGSEA, weighted gene co-expression network analysis (WGCNA), and univariate Cox regression analysis were used to screen promising candidates. Using a random forest algorithm and combinatorial screening methods, a prognostic gene signature associated with MYC targets V1 and V2 was developed. The prognostic value of the risk score based on the gene signature was assessed in a training set (TCGA) and an independent validation set (GSE17918). Table 1 summarizes patient information from the TCGA and GEO cohorts.

**Table 1:** Clinical information of patients with sarcoma included in this study.

**Characteristic**		**TCGA (*n* = 256)**	**GSE17118 (*n* = 57)**
Status	Alive	158 (61.72%)	27 (47.37%)
	Dead	98 (38.28%)	30 (52.63%)
Age	≤60	128 (50.00%)	40 (70.18%)
	>60	128 (50.00%)	17 (29.82%)
Gender	Female	139 (54.30%)	31 (54.39%)
	Male	117 (45.70%)	26 (45.61%)
Race	Asian	5 (1.95%)	
	Black	18 (7.03%)	
	White	224 (87.50%)	
	Not reported	9 (3.52%)	
Grade	G1 and G2		28 (49.12%)
	G3		18 (31.58%)
	Unknown		11 (19.30%)

### MYC targets V1 and V2 were identified as critical survival factors in sarcoma

**Figure 2:**
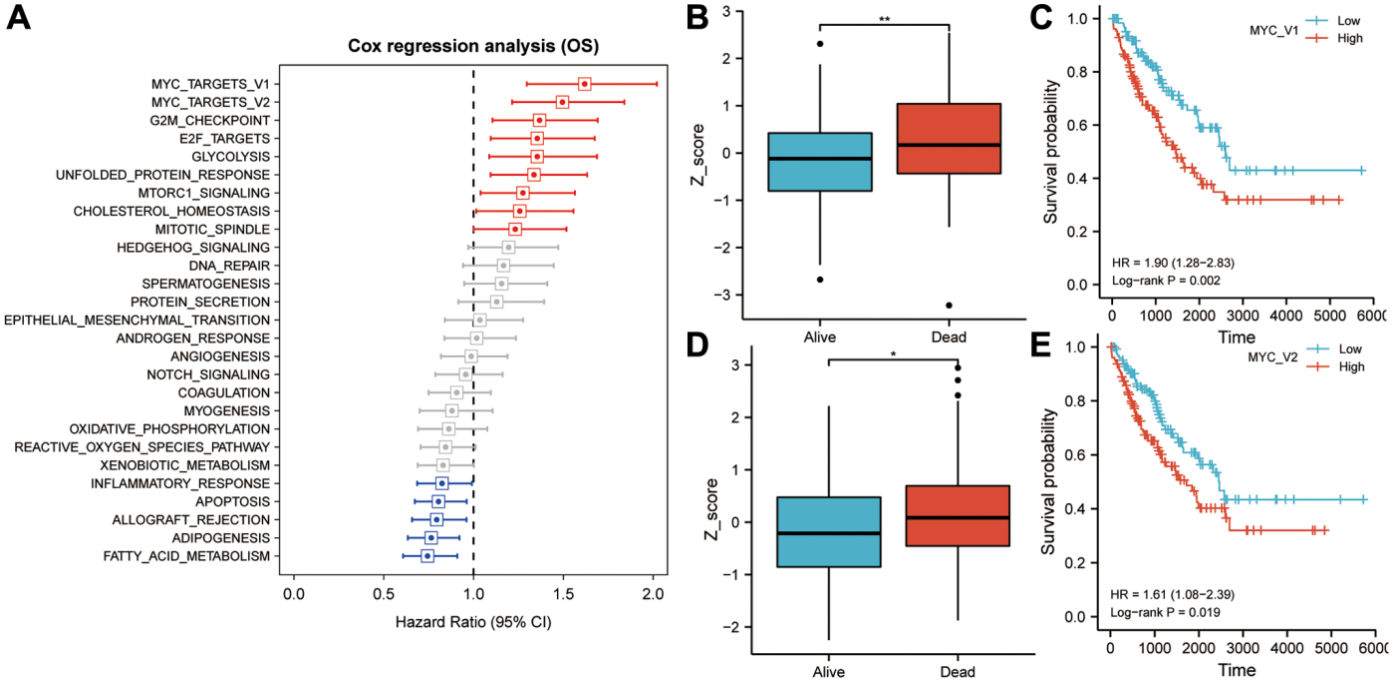
**MYC targets V1 and V2 play an important role in overall survival in sarcoma patients.** (**A**) In univariate Cox regression analysis, MYC targets V1 and V2 exhibited the highest prognostic significance for sarcoma patients. (**B**) Patients who died during follow-up had significantly higher Z-scores for MYC targets V1 than those who survived. (**C**) In Kaplan-Meier analysis, patients with higher Z-scores for MYC targets V1 exhibited poorer OS. (**D**) Patients who died during follow-up had significantly higher Z-scores for MYC targets V2 than those who survived. (**E**) In Kaplan-Meier analysis, patients with higher Z-scores for MYC targets V2 exhibited poorer OS. Abbreviation: OS: overall survival. Asterisks indicate statistical significance at: ^*^p < 0.05 and ^**^p < 0.01.

TCGA-SARC cohort overall survival data and ssGSEA scores were used to calculate and rank the Cox coefficients for cancer hallmarks. As a result of univariate analysis, MYC targets V1 and V2 had a greater impact on sarcoma patient survival than the G2M checkpoint, E2F targets, glycolysis, unfolded protein response, mtorc1 signaling, cholesterol homeostasis, mitotic spindle and other cancer hallmarks (Figure 2A). A higher Z-score was observed for MYC targets V1 and V2 for patients who died during the follow-up period, as shown in Figure 2B, 2D. The median Z-score was used as the cut off value to separate 256 TCGA-SARC sarcoma patients into high- and low-risk groups. In the high MYC targets V1 Z-score group, overall survival rates were lower than that in the low MYC targets V1 Z-score group (HR = 1.90, *P* = 0.002; Figure 2C). Similarly, in the high MYC targets V2 Z-score group, overall survival rates were lower than that in the low MYC targets V2 Z-score group (HR = 1.61, *P* = 0.019; Figure 2E).

### WGCNA screening identified genes that correlated strongly with MYC targets V1 and V2

**Figure 3:**
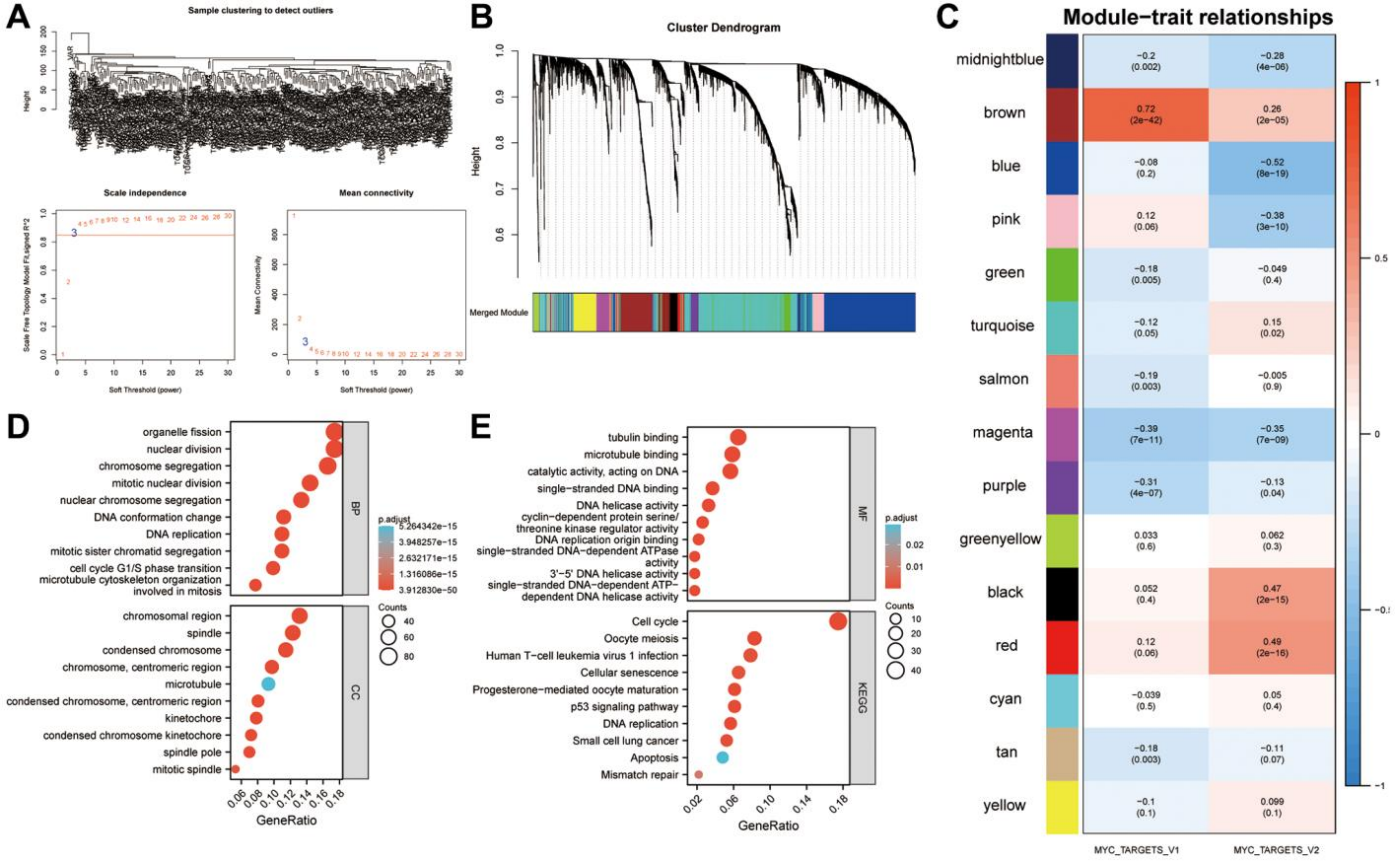
**Identification of a gene signature associated with the MYC targets V1 and V2.** (**A**) Identification of DEGs in sarcoma. The variance of expression values for each gene in sarcoma patients (*n* = 256) was calculated using |log2FC|>1 and FDR < 0.05. (**B**) An analysis of the top 6000 DEGs was performed to identify non-grey modules in the WGCNA network. (**C**) Modules associated with key cancer hallmarks were constructed. The brown module had a higher correlation with MYC targets V1 (r = 0.72, *P* < 0.001) and V2 (r = 0.26, *P* < 0.001). (**D**, **E**) All genes from the brown module were analyzed using GO and KEGG. Abbreviations: WGCNA: weighted gene co-expression network analysis; DEG: differentially expressed genes; FDR: false discovery rate.

A total of 256 SRAC patients’ expression values were calculated and then the top 6000 genes by variance were selected to construct the WGCNA network (Figure 3A). In order to generate non-grayscale modules, the transcriptome analysis data of 6000 genes was used with β = 3 as the optimal soft threshold. Co-expression modules related to key cancer hallmarks were then developed (Figure 3B). A total of 15 modules were obtained by WGCNA network. As shown in Figure 3C, the brown module showed strong correlation with MYC targets V1 (r = 0.72, *P* < 0.001) and V2 (r = 0.26, *P* < 0.001). Following this, all the genes of the brown module were subjected to GO and KEGG analyses (Figure 3D, 3E).

### A prognostic gene signature relating to MYC targets V1 and V2

**Figure 4:**
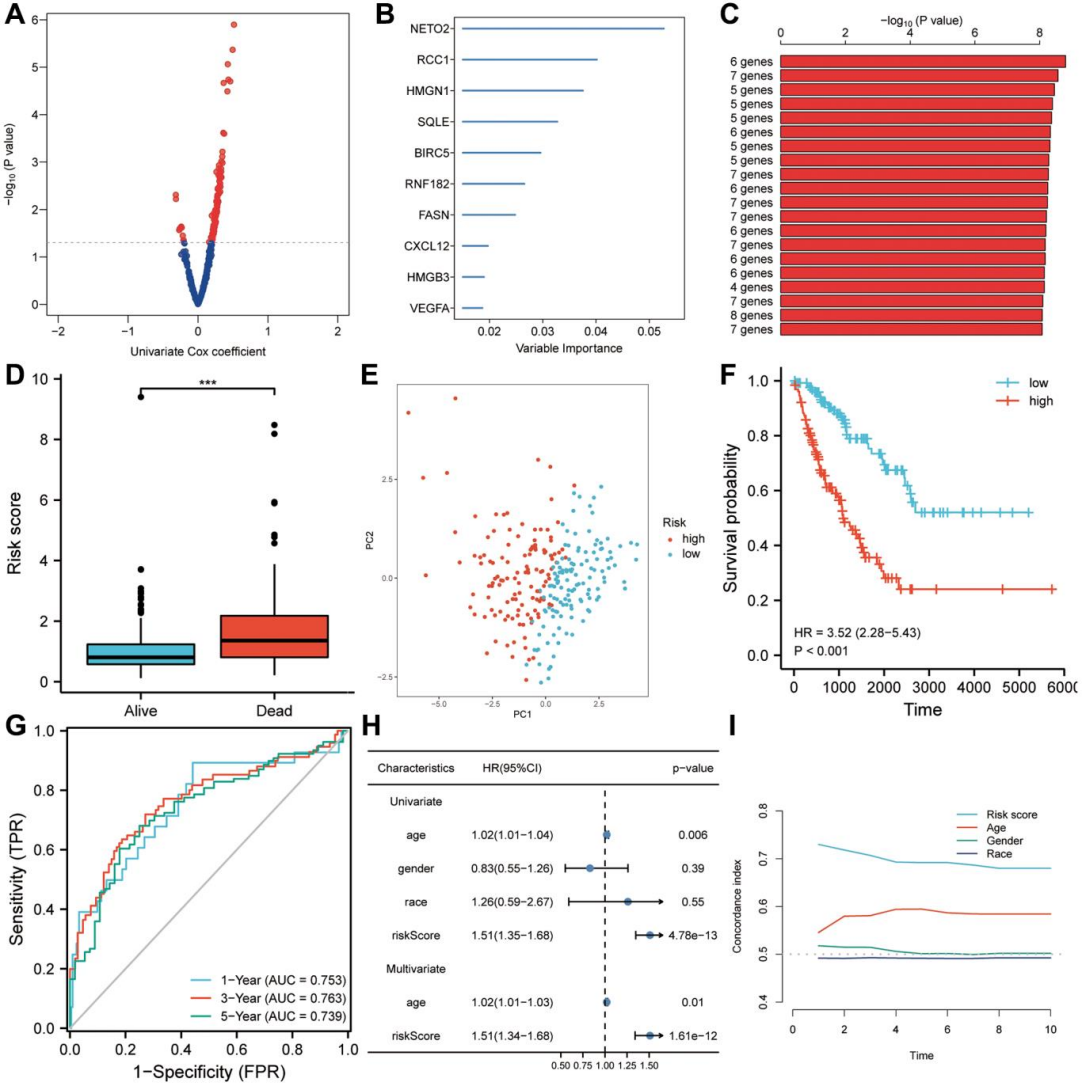
**Risk scores are predictive of poor survival in the TCGA-SARC set.** (**A**) A univariate Cox analysis determined that 147 candidates from the brown module were associated with prognosis for sarcoma patients (*P* < 0.05). (**B**) The top ten genes with the highest genetic significance were selected using random forest. (**C**) A survival prediction model was constructed using a gene combination with a relatively small number of genes and a significant *P* value. (**D**) A higher risk score was observed in patients who died during follow-up than in people who survived. (**E**) A Kaplan-Meier analysis revealed poor overall survival in the high-risk score group. (**F**) In a principal component analysis, risk score was found to be a useful tool for evaluating a patient’s prognosis with sarcoma. (**G**) Overall survival was predicted well by risk scores (AUC > 0.73) in patients with sarcomas. (**H**) Risk score was found to be an independent risk factor for OS in both univariate and multivariate Cox regression analyses. (**I**) A C-index analysis concluded that the risk score had significantly more predictive power than any other clinical characteristic. Abbreviations: HR: hazard ratio; ROC: receiver operating characteristic. Statistical significance is indicated by an asterisk: ^***^p < 0.001.

In a univariate Cox regression analysis, 147 genes from the brown module were found to be most significantly associated with prognosis in patients with sarcoma (Figure 4A). A random forest was used to rank the importance of candidate genes, and the 10 most significant genes were screened (Figure 4B). After selecting a gene combination with fewer genes and more significant *p*-values from multiple combinations of 10 genes, a survival risk model was constructed (Figure 4C). Finally, a prognostic model for sarcoma patients was constructed using six hub genes: risk score = 0.05 × VEGFA + 0.25 × HMGB3 + 0.22 × FASN + 0.40 × RCC1 + 0.46 × NETO2-0.10 × BIRC5. A significant difference was found between the risk scores of patients who died during follow-up and those who survived (Figure 4D). Risk score has been shown to be a useful tool for evaluating sarcoma patients’ prognoses based on principal component analysis (Figure 4E). The overall survival of the high-risk score group was lower (HR = 3.52, *P* < 0.001, Figure 4F). As shown by the ROC curve, the AUCs of the risk score were 0.753, 0.763, and 0.739 for predicting 1-year, 3-year, and 5-year survival, respectively (Figure 4G). So the risk score appeared to be a good predictor of survival in patients with sarcoma. Further, univariate and multivariate Cox regression analyses showed that age (HR = 1.02, *p* = 0.01) and risk score were independent predictors of overall survival (Figure 4H). According to a subsequent analysis of the C-index, the risk score predicted survival more accurately than clinical factors such as age, gender and race (Figure 4I).

### Independent validation of the six-gene signature in sarcoma

**Figure 5:**
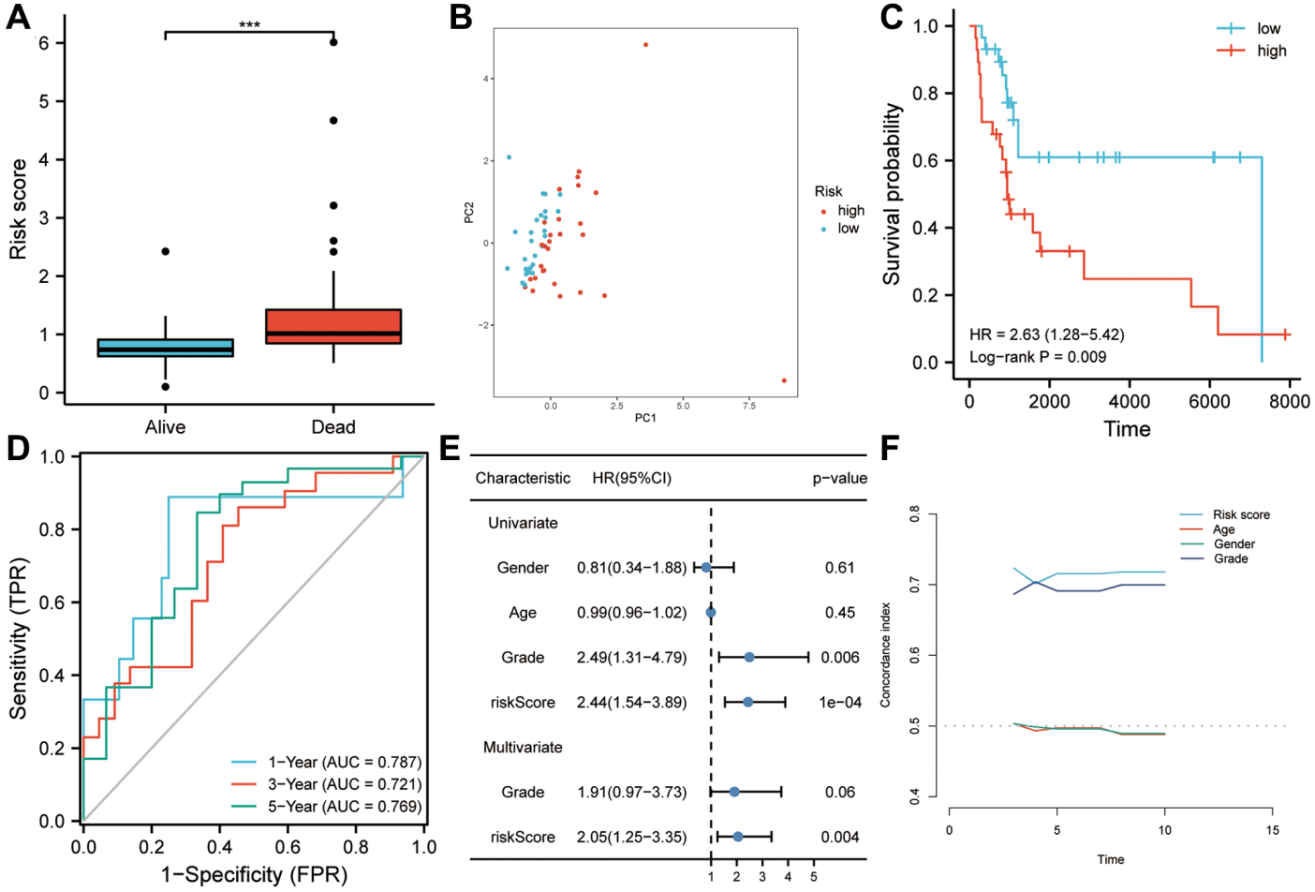
**Using GSE17118 as a validation dataset for the risk model.** (**A**) Patients who died during follow-up had a higher risk score than those who survived. (**B**) Risk score was found to be a useful tool to assess the prognosis of sarcoma patients based on principal component analysis. (**C**) Patients with higher risk scores had a worse overall survival according to Kaplan-Meier analysis. (**D**) As shown in the ROC curve, risk scores had good predictive value for survival in sarcoma patients (AUC > 0.72). (**E**) Risk score was found to be an independent risk factor for OS in both univariate and multivariate Cox regression analyses. (**F**) The risk score’s predictive power was significantly higher than that of other clinical characteristics, according to the C-index analysis. Abbreviations: HR: hazard ratio; ROC: receiver operating characteristic. Statistical significance is indicated by an asterisk: ^***^p < 0.001.

In order to validate the robustness of the prognostic six-gene signature associated with MYC targets V1 and V2, an independent external cohort (GSE17118) was utilized. A significant difference between patients who died during follow-up and those who survived is demonstrated in Figure 5A (*P* < 0.001). Figure 5B shows that the risk score may provide a new dimension for assessing sarcoma prognosis. Based on Kaplan-Meier analysis, patients with high-risk scores had a lower overall survival rate (*P* < 0.01; Figure 5C). In the ROC curve, the accuracy of the risk score for the prediction of 1-, 3-, and 5-year survival was 0.787, 0.721, and 0.769, respectively, showing that this model is effective in predicting survival (Figure 5D). Moreover, Cox regression analysis of univariate and multivariate data showed that risk score can be an independent predictor of overall survival (HR = 2.05, *p* = 0.004; Figure 5E). In the study of the C-index, the risk score was significantly better at predicting survival than any clinicopathological characteristic, such as age, gender and grade (Figure 5F).

### A two-way survival analysis based on correlations between risk scores and cancer hallmarks

**Figure 6:**
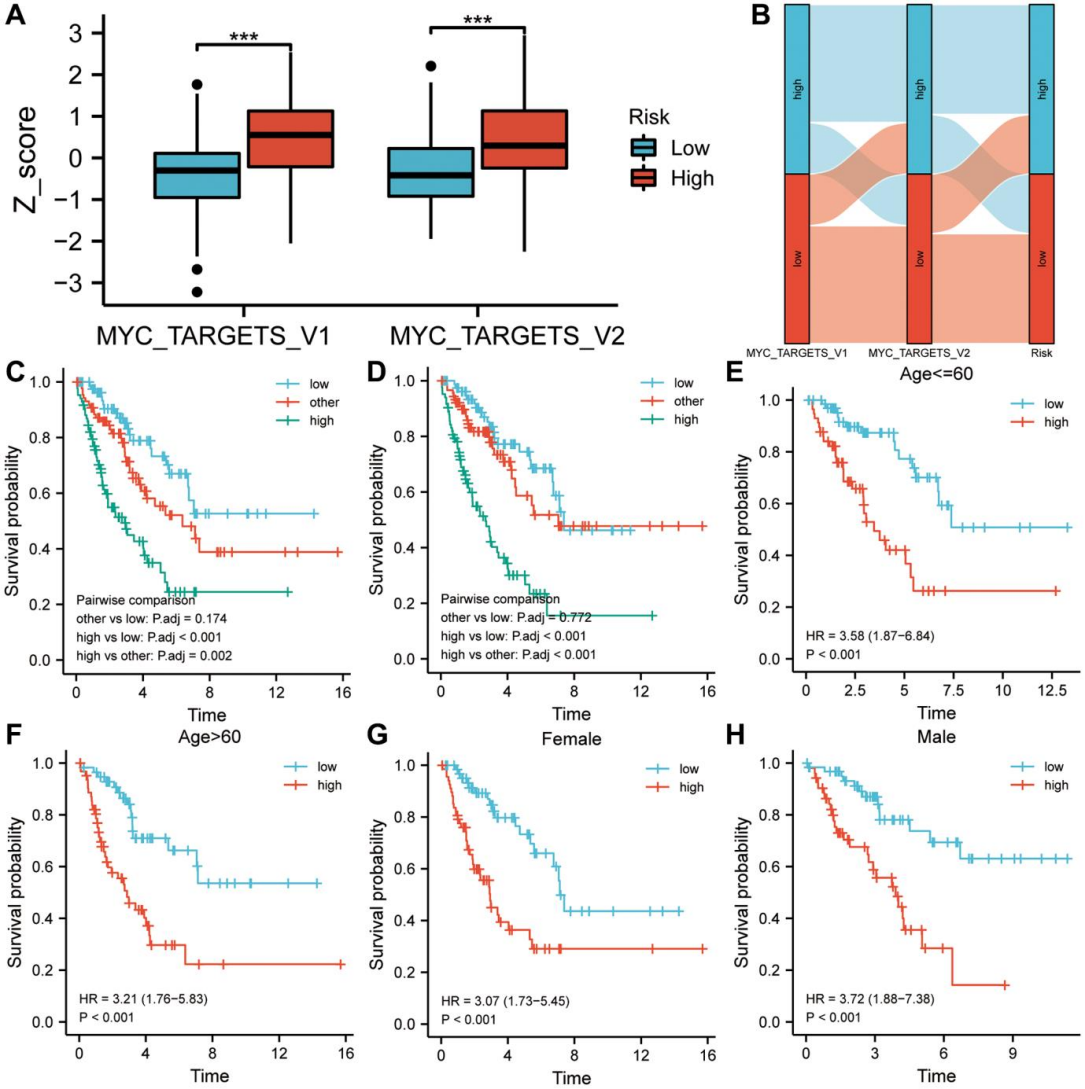
**A two-factor analysis of survival incorporating cancer hallmarks and risk scores.** (**A**) MYC targets V1 and V2 of the high-risk group showed significantly higher Z-scores than those of the low-risk group. (**B**) A correlation analysis was conducted among the cancer hallmarks, the risk score, and the survival status of sarcoma patients. (**C**) High MYC targets V1 & high-risk score predicted a worse prognosis in a two-factor survival analysis. (**D**) High MYC targets V2 & high-risk score predicted a worse prognosis in a two-factor survival analysis. (**E**–**H**) In various subgroups based on clinicopathological characteristics, the risk score was a biomarker of poor prognosis. Abbreviation: HR: risk ratio.

In Figure 6A, both MYC targets V1 and V2 Z-scores were higher in the high-risk group. High-risk groups had higher proportions of high Z-scores on MYC targets V1 and V2 than low-risk groups, as shown in Figure 6B. The results of the two-way survival analysis combined with cancer hallmarks indicated that sarcoma patients with high-risk-scores and high MYC targets Z-scores showed the lowest overall survival rates (Figure 6C, 6D). Figure 6E–6H illustrates how the six-gene signature can distinguish high-risk patients with poor prognoses based on clinical characteristics such as age and gender (*P* < 0.001).

### Analysis of the correlation between the risk score and the tumor immune microenvironment

**Figure 7:**
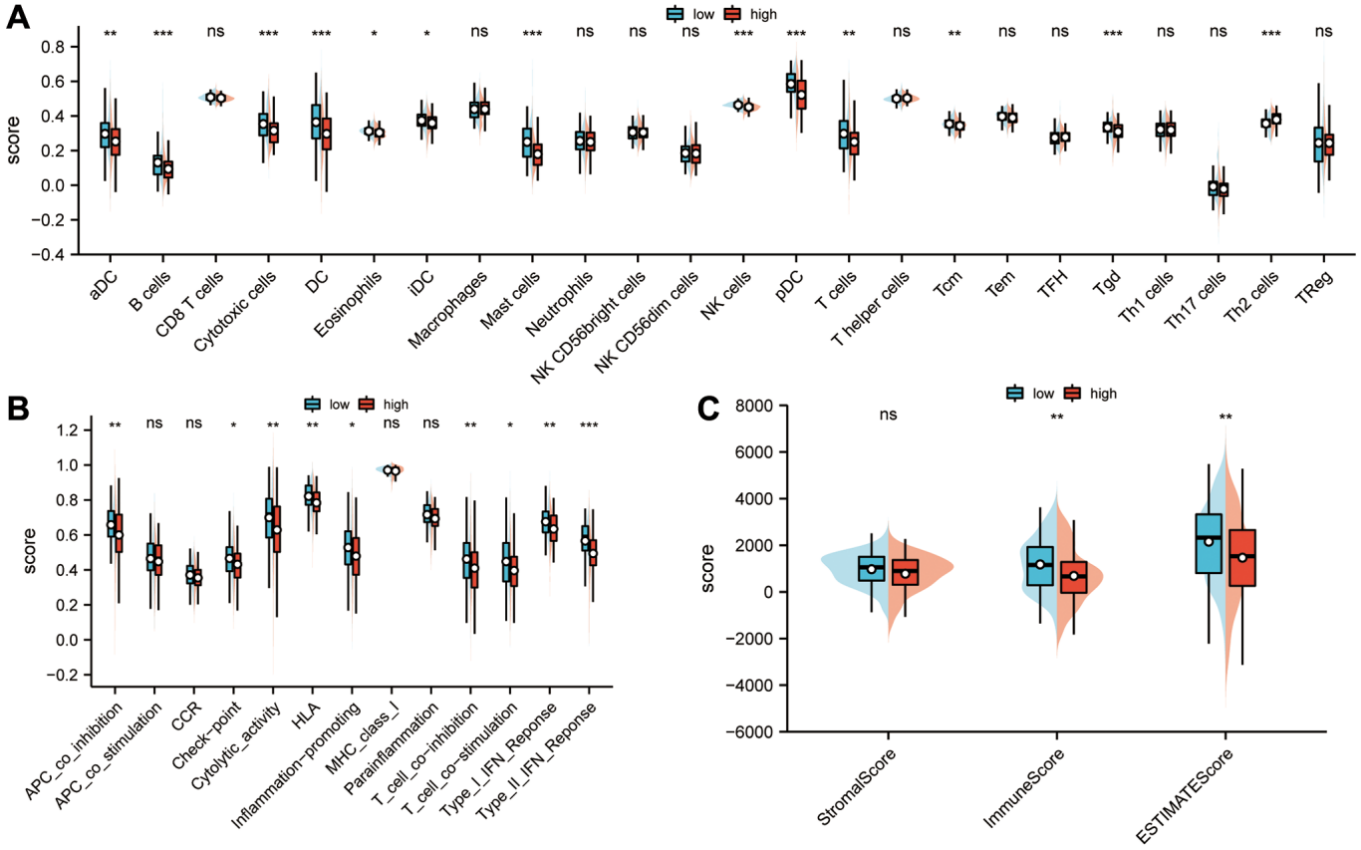
**Analysis of the correlation between sarcoma risk score and tumor immune infiltration.** (**A**) The degree of immune infiltration and immune function status of high- and low-risk groups were calculated using ssGSEA. In the low-risk group, there was a higher degree of immune infiltration of aDCs, B cells, cytotoxic cells, DCs, eosinophils, iDCs, mast cells, NK cells, pDCs, T cells, Tcm, and Tgds than in the high-risk group, while Th2 cells were more infiltrated in the high-risk group than those in the low-risk group (*P* < 0.05). (**B**) In comparison with the high-risk group, the low-risk group had higher scores for APC co-inhibition immune function scores, HLA, inflammation-promoting, check-point, cytolytic activity, T cell co-inhibition, T cell co-stimulation, type I IFN response and type L IFN response (*P* < 0.05). (**C**) An ESTIMATE algorithm was used in calculating stromal scores, immune scores, and estimation scores. Abbreviation: ESTIMATE: estimation of stromal and immune cells in malignant tumor tissues using expression data. Statistical significance is indicated by an asterisk: ^*^p < 0.05; ^**^p < 0.01 and ^***^p < 0.001.

Based on ssGSEA algorithms, immune cell infiltration profiles and immune function status were determined in high- and low-risk groups. The results suggested that immune infiltration degrees of aDC, B cells, cytotoxic cells, DC, eosinophils, iDC, mast cells, NK cells, pDC, T cells, Tcm and Tgd in the low-risk group were higher than those in the high-risk group, while a higher degree of immune infiltration of Th2 cells was observed in the high-risk group (*P* < 0.05; Figure 7A) than in the low-risk group. Compared to the high-risk group, the low-risk group scored higher on type I IFN response, type L IFN response, T cell co-stimulation, T cell co-inhibition, inflammation-promoting, HLA, cytolytic activity, check-point and APC co-inhibition (*P* < 0.05; Figure 7B). The ESTIMATE algorithm was also used to calculate the abundance of immune cells, stromal cells, and tumor cells in the sarcoma microenvironment. A significant difference was found between the low-risk and high-risk groups in stromal scores, immune scores, and estimates of sarcoma (*P* < 0.01; Figure 7C).

### Using risk scores to predict drug therapy

**Figure 8:**
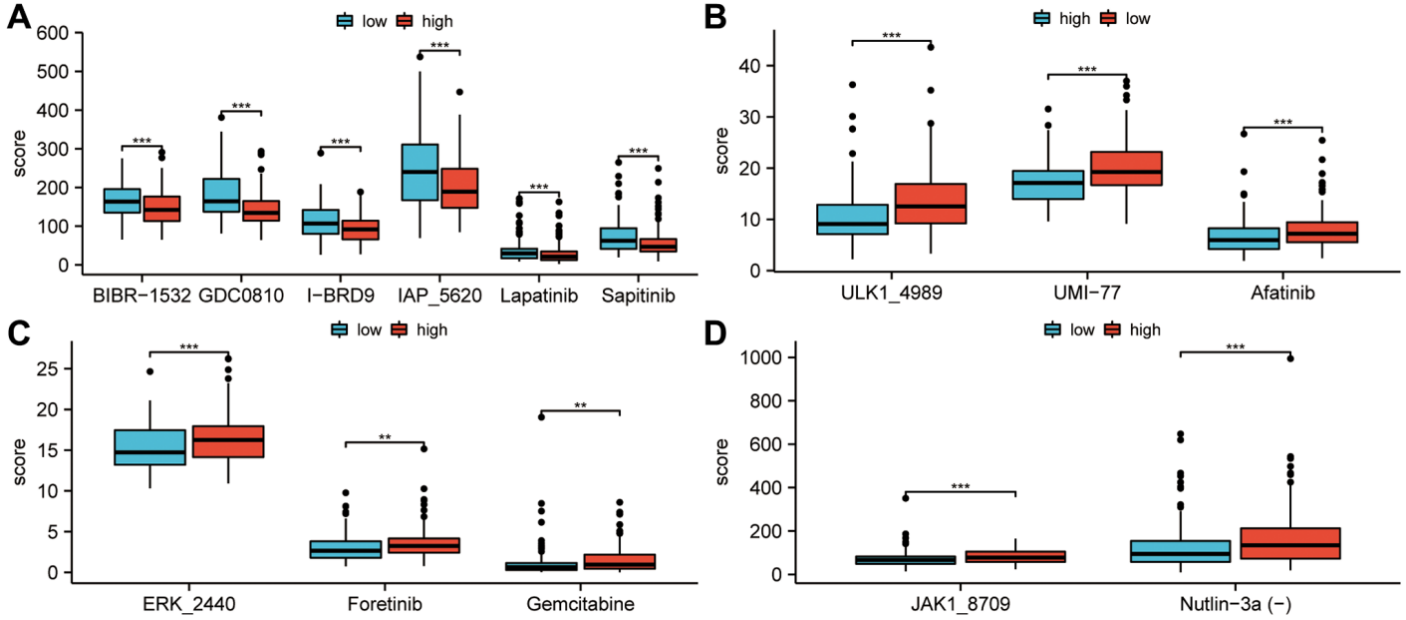
**Study of the efficacy of drug therapy for low-risk and high-risk patients.** (**A**) In the low-risk groups, treatment scores for BIBR-1532, GDCO-810, I-BRD9, IAP-5620, Lapatinib and Sapitinib were higher than in the high-risk groups. (*P* < 0.001). (**B**) In the low-risk groups, treatment scores for ULK-14989, UMI-77, and Afatinib were higher than in the high-risk groups (*P* < 0.001). (**C**) Foretinib, Gemcitabine, and ERK-2440 treatment scores in the high-risk groups were higher than in the low-risk groups (*P* < 0.01). (**D**) JAK1-8709 and Nutlin-3a(-) treatment scores were higher in high-risk groups than in low-risk groups (*P* < 0.001). Statistical significance is indicated by an asterisk: ^*^p < 0.05; ^**^p < 0.01 and ^***^p < 0.001.

For the purpose of selecting effective drugs for sarcoma patients, drugs with different treatment predictions for high and low risk groups were explored. As shown in Figure 8A, BIBR-1532, GDCO810, I-BRD9, IAP5620, Lapatinib, and Sapitinib scored higher in the low-risk groups than in the high-risk groups (*P* < 0.001). Low-risk groups achieved significantly higher drug treatment scores with ULK 14989, UMI 77, and Afatinib (*P* < 0.001; Figure 8B). The treatment scores for ERK-2440, Foretinib, and Gemcitabine in the high-risk groups were higher than those in the low-risk groups (*P* < 0.01; Figure 8C). Additionally, a higher treatment score for JAK1-8709 and Nutlin-3a(-) was observed for high-risk groups (*P* < 0.001; Figure 8D).

### Analyzing the susceptibility of six hub genes to drugs

**Figure 9:**
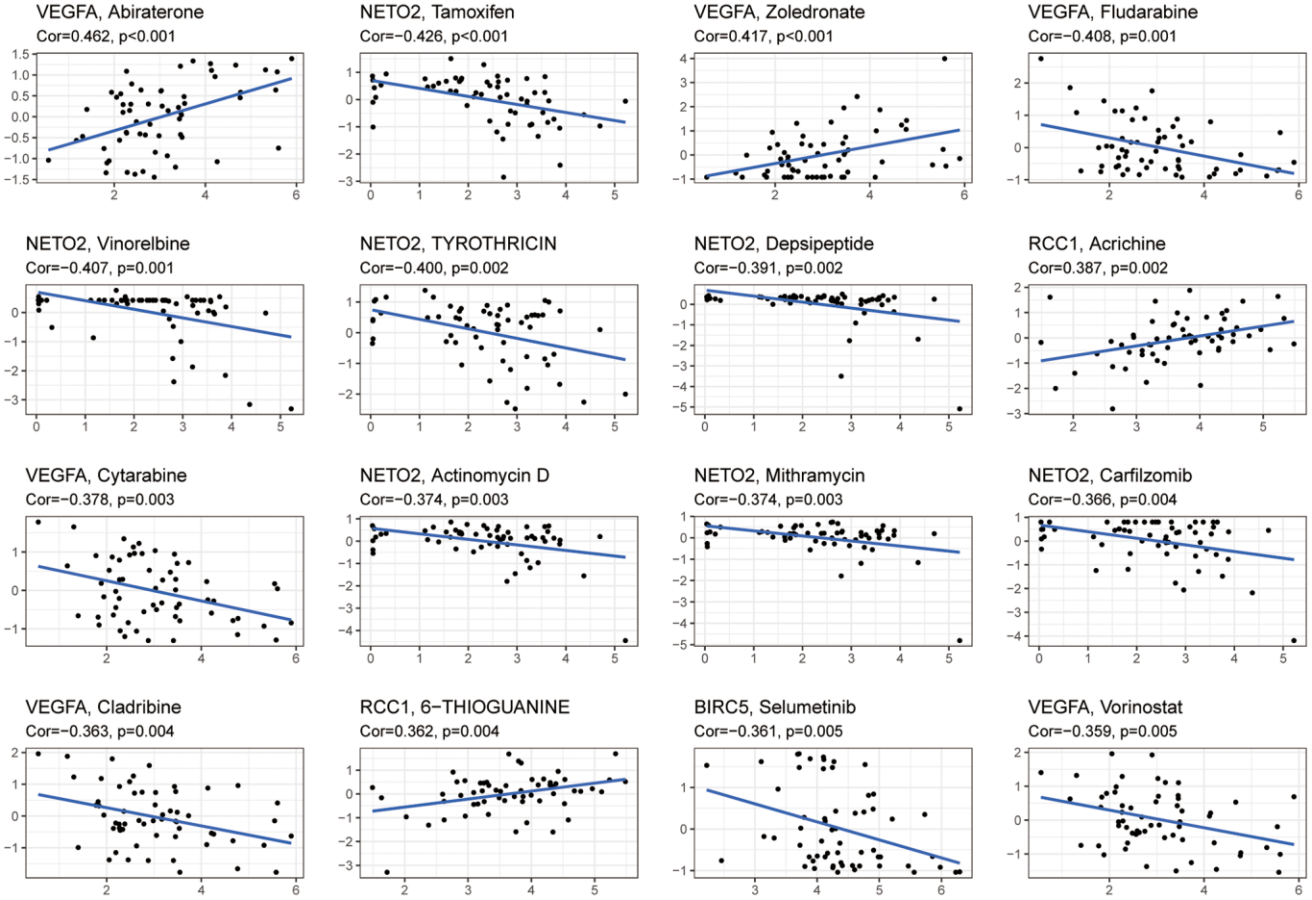
**An analysis of six hub genes for drug sensitivity.** Drug susceptibility to Abiraterone and Zoledronate was positively correlated with VEGFA expression, while Fludarabine, Cytarabine, Cladribine, and Vorinostat were negatively correlated. Drug susceptibility to Tamoxifen, Vinorelbine, Tyrothricin, Depsipeptide, Actinomycin, Mithramycin, and Carfilzomib was negatively correlated with NETO2 expression. Drug sensitivity to Acrichine and 6-Thioguanine was positively correlated with RCC1 expression. A negative correlation was found between Selumetinib sensitivity and BIRC5 expression. Abbreviation: Cor: correlation coefficient.

CellMiner™ database was used to analyze drug sensitivity for the six hub genes. Results showed a positive correlation between VEGFA expression and Abiraterone and Zoledronate drug susceptibility, as well as a negative correlation with Fludarabine, Cytarabine, Cladribine, and Vorinostat. There was a negative correlation between NETO2 expression and drug susceptibility to Tamoxifen, Vinorelbine, Tyrothricin, Depsipeptide, Actinomycin, Mithramycin and Carfilzomib. A positive correlation was observed between the expression of RCC1 and the drug sensitivity of acricine and 6-thioguanine. There was an inverse correlation between BIRC5 expression and Selumetinib susceptibility (Figure 9).

### Gene differentially expressed and enrichment analysis

**Figure 10:**
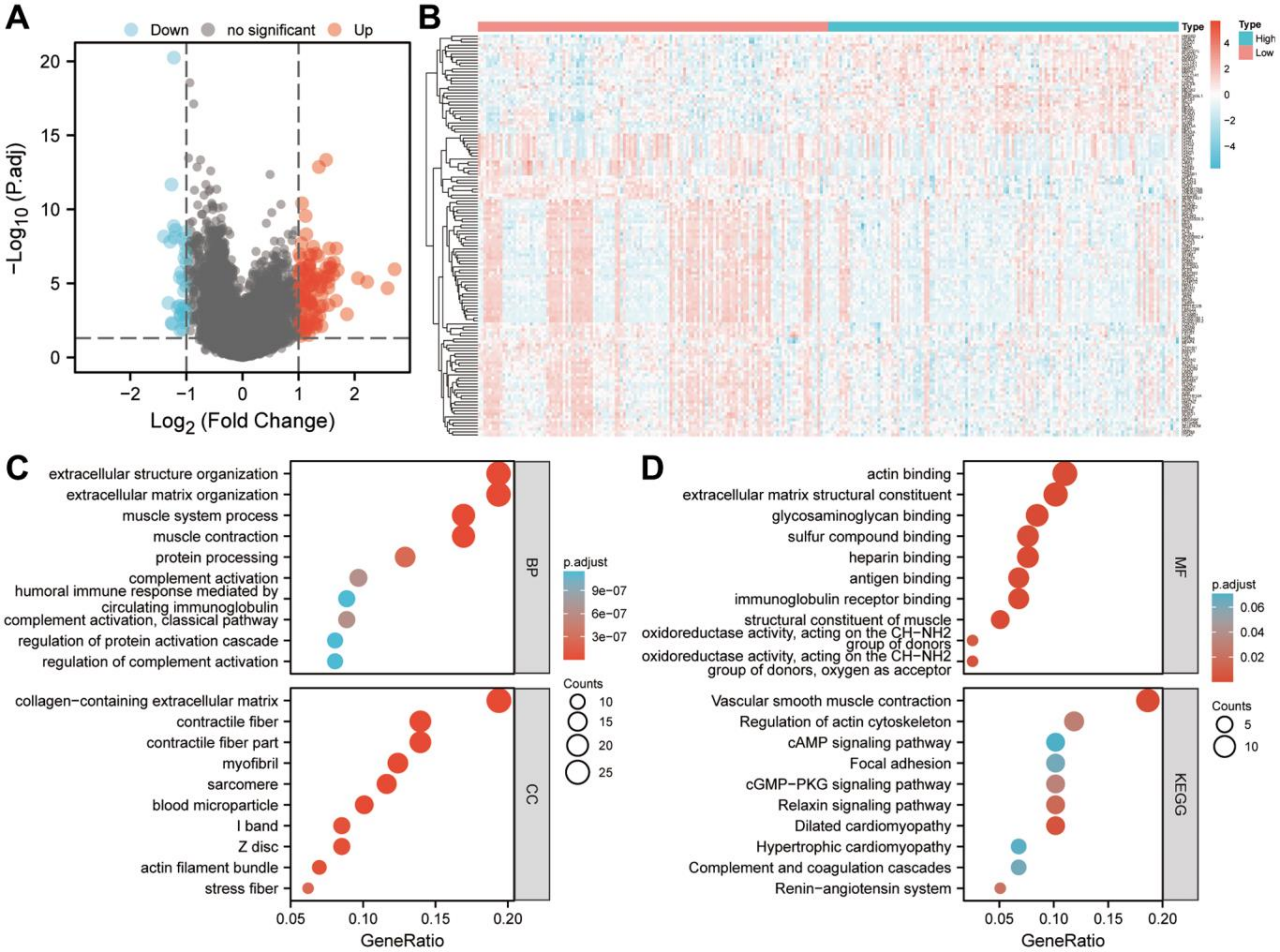
**Based on the risk score, enrichment analysis is performed.** (**A**) Determination of significantly different DEGs between low-risk and high-risk groups (|log2FC|>1 and FDR < 0.05). (**B**) DEG expression profiles in high-risk and low-risk groups shown as a heatmap. (**C**, **D**) Analysis of DEGs between high-risk and low-risk groups using GO and KEGG. Abbreviations: BP: biological process. CC: cell component. MF: molecular function. DEGs: differentially expressed genes. FDR: false discovery rate.

256 patients were divided into high- and low-risk groups according to the median of their risk scores in the TCGA-SARC cohort. Genes with differential expression were identified between the two groups. We then examined significantly enriched GO terms and KEGG pathways associated with DEGs. A total of 134 DEGs in sarcoma were identified using log2FC > 1 and *P* < 0.05 as thresholds (Figure 10A). As shown in Figure 10B, a heatmap showed the differential expression of DEGs between high-risk and low-risk groups. Figure 10C shows that 134 DEGs were significantly enriched in biological processes such as the organization of extracellular structures, organization of extracellular matrix, process of muscle contraction, processing of proteins, activation of complement, a humoral immune response mediated by circulating immunoglobulins, complement activation, regulation of protein activation cascades and complement activation. Among the cell components that were significantly enriched were collagen-containing extracellular matrix, contractile fiber, contractile fiber part, myofibril, sarcomere, blood microparticles, I band, Z disc, actin filament bundle and stress fiber. In addition, the significantly enriched molecular functions in Figure 10D included the binding of actin, extracellular matrix structural components, glycosaminoglycans, sulfur compounds, heparin, antigens, and immunoglobulin receptors, oxidoreductase activity acting on the CH-NH2 group of donors and oxidoreductase activity acting on the CH-NH2 group of donors and oxygen as acceptor. Moreover, significant KEGG pathways were enriched for vascular smooth muscle contraction, cytoskeleton regulation, CAMP signaling, focal adhesion, cGMP-PKG signaling pathway, relaxin signaling pathway, dilated cardiomyopathy, hypertrophic cardiomyopathy, the complement and coagulation cascades, and Renin-angiotensin system. So tumorigenesis and development may be influenced by the six-gene signature.

### Nomogram construction for predicting survival in sarcoma patients

**Figure 11:**
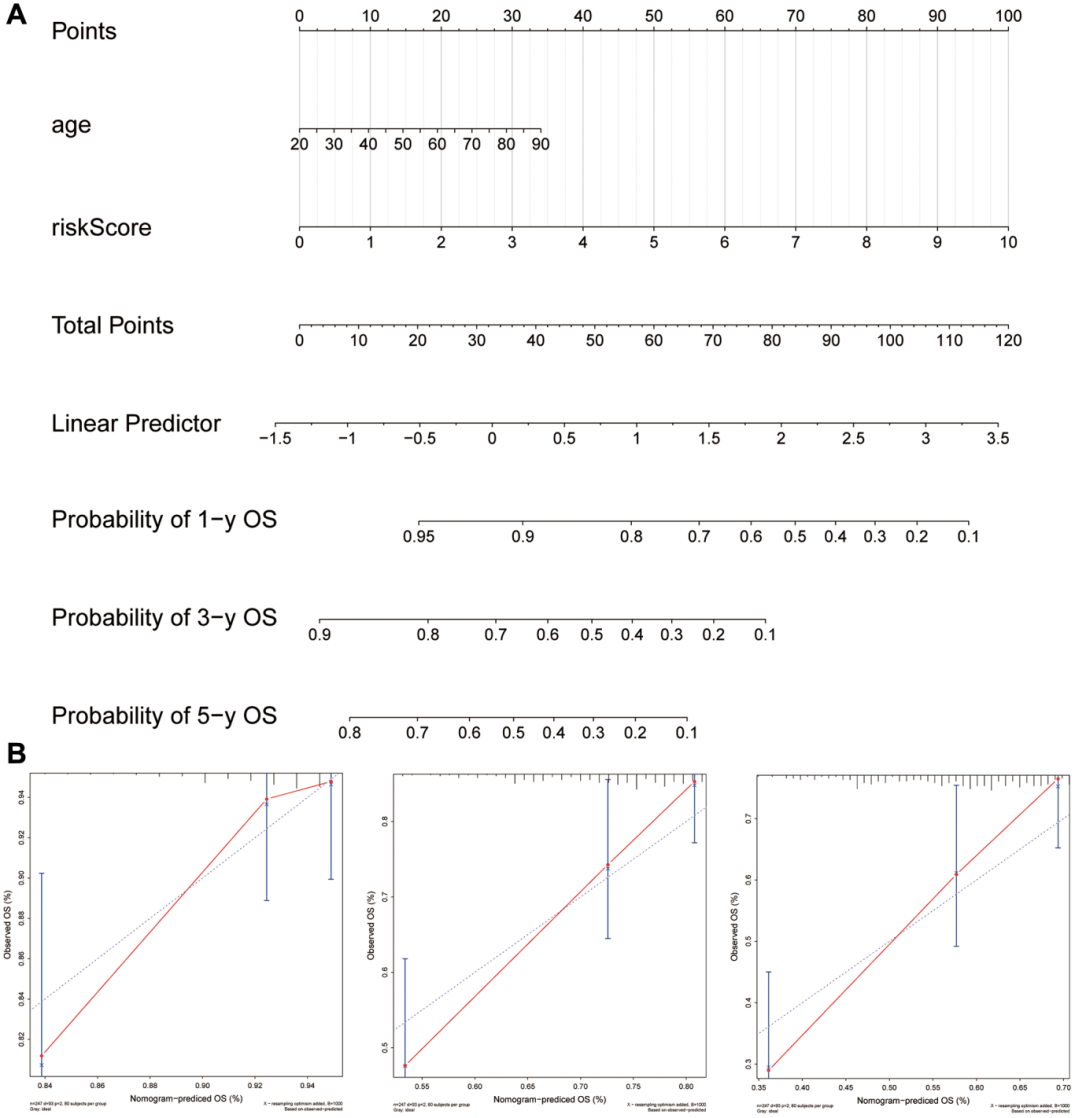
**Using the six-gene signature, a nomogram was developed for clinical practice in patients with sarcoma.** (**A**) A six-gene signature was combined with age to create a nomogram. (**B**) Observations and calibration plots showing similar survival probabilities for 1, 3, and 5 years.

With the six-gene signature and patient age combined, a clinically useful nomogram was constructed (Figure 11A). By analyzing calibration curves (Figure 11B), the predictive power of this nomogram was verified. According to the results, the predicted 1-, 3-, 5- and 10-year survival probabilities were mostly in agreement with what actually occurred.

## DISCUSSION

Sarcomas have a poor prognosis and inadequate treatment options. For this reason, sarcoma biomarker screening is of great importance for determining prognosis and identifying possible therapeutic targets. The aim of this study was to build a prognostic gene signature using integrated bioinformatics techniques based on the cancer hallmarks. Firstly, a ssGSEA and Cox-PH analysis identified MYC targets V1 and V2 as the highly associated cancer hallmarks with overall survival in sarcoma patients. For further analysis, all genes associated with MYC targets V1 and V2 were extracted. Following this, WGCNA, COX univariate regression analysis, random forest, and combination screening techniques were used to develop a prognostic six-gene signature for patients with sarcomas. Subsequently, an independent sarcoma cohort was used to validate the prognostic values of the gene signature. In addition, the six-gene signature was correlated with the tumor immune microenvironment. Thus, this six-gene signature could provide independent prognostic information.

Prognostic assessment is essential for clinical treatment of sarcoma patients. Sarcoma patients’ prognosis is influenced by clinical characteristics, such as age, tumor size, metastasis, tumor grade, primary site, and histological response [26, 27]. In addition, patients with sarcoma have a high mortality rate when their absolute lymphocyte counts are abnormally low at diagnosis [28]. A neutrophil-lymphocyte ratio and platelet-lymphocyte ratio were considered independent prognostic factors in sarcoma patients following surgery [29]. In terms of gene signatures, an immune-related five-gene signature based on MYBL2, FBN2, TSPAN7, GCSH, and DDX39B is a prognostic biomarker for sarcoma patients [30]. The presence of hypoxia in sarcoma is associated with metastasis and poor prognosis. And a hypoxia-related biomarker CAIX has prognostic value in sarcoma [31]. A hypoxia-related signature based on 24 genes is helpful in evaluating the prognosis of sarcoma patients [32]. Sarcomas that are at high risk can be identified using the genome grading index and sarcoma complexity index [33, 34]. In addition, many immune-related genetic signatures were identified. For example, an immune-related prognostic seven-gene signature was negatively associated with tumor purity and positively associated with levels of B cell, CD4+ T cell, and CD8+ T cell infiltration [35]. Moreover, an eleven-gene signature has been shown to be associated with the prognosis of Ewing’s sarcoma [36]. In general, previous studies have constructed some prognostic genetic markers of sarcoma based on phenotypes such as immunity [37], apoptosis [38], pyrodeath [39], and hypoxia [40]. Different from previous studies, this study constructs a new sarcoma prognostic gene marker based on MYC targets V1 and V2, which adds new content to the research in this field.

The six-gene signature constructed in this study included six hub genes, VEGFA, HMGB3, FASN, RCC1, NETO2 and BIRC5. VEGFA is associated with protein homodimerization activity and protein heterodimerization activity. Previous studies have shown that the expression of soluble VEGFA subtypes can increase the metastasis of fibrosarcoma through multiple mechanisms [41]. Inhibition of VEGFA expression in human Ewing sarcoma cells can inhibit cell growth and tubule formation [42]. NETO2 is associated with ionotropic glutamate receptor binding. NETO2 can act as an oncogene for osteosarcoma by activating the PI3K/AKT pathway [43]. BIRC5 is involved in pathways associated with Class I MHC-mediated antigen processing and presentation and protein metabolism. The overexpression of BIRC5 in Ewing sarcoma was an independent adverse prognostic factor [44]. In addition, HMGB3 is associated with RNA binding and double-stranded DNA binding. FASN is associated with RNA binding and identical protein binding. RCC1 is related with pathways including DNA Damage and Validated targets of C-MYC transcriptional activation. As far as we know, the function of HMGB3, FASN and RCC1 in sarcoma has not been reported.

There has been evidence that the tumor microenvironment influences the prognosis of sarcomas in previous studies. For example, CD20/MS4A1 expression can be used as a prognostic tool for sarcoma survival prediction, but not in patients with a markedly immunosuppressive tumor microenvironment [45]. An increase in lymphocytic infiltration was associated with a greater overall survival in non-translocation-related sarcomas. It was found that expression of PD-1 and CD56 was associated with poorer survival overall [46]. In synovial sarcoma, patients with higher CD8+ or FOXP3+ lymphocyte infiltration were associated with good overall survival, whereas patients with higher CD163+ macrophage infiltration had significantly lower overall and progression-free survival [47]. Sarcoma is a heterogeneous tumor with multiple subtypes. Although a subset of sarcomas responds to immune checkpoint blockade with PD-1-targeted agents, most subtypes require new immunotherapies or combination therapies [48]. Sarcoma patients can benefit from immune checkpoint inhibitors, adoptive cell therapy, and cancer vaccines [49]. Responses to immune checkpoint blockade therapy vary widely among various subtypes of patients. The immune checkpoint blockade therapy was more effective for patients with high B-cell infiltration and tertiary lymphoid histology [50]. Immune infiltration in sarcoma has been shown to correlate with response to pembrolizumab treatment [51]. Retroperitoneal liposarcoma patients with high PD-1/PD-L1 expression have a poor prognosis, while patients with tertiary lymphoid structure have better disease-free survival [52]. In the present study, high-risk patients also had more TH2 cells, which play an important role in tumor survival, while low-risk patients had more CD8 and NK cells, suggesting that immunosuppressive therapies could target the six-gene signature which is associated with the immune microenvironment.

This study has several limitations. Although the present study used various independent datasets for mutual validation to verify the prognostic values of this six-gene signature. However, *in vitro* experiment is still an important step to further confirm the prognostic values of this six-gene signature. In addition, this is a retrospective study, so it is necessary to verify the robustness of this six-gene signature in future prospective studies.

In conclusion, this study not only confirms the prognostic significance of MYC targets V1 and V2, but also established a new survival model based on six hub genes including VEGFA, HMGB3, FASN, RCC1, NETO2 and BIRC5. We believe this six-gene signature is an independent biomarker for survival prediction in sarcoma patients. In addition, whether these six hub genes can be used as potential targets for targeted therapy is also worthy of further exploration.
